# HOPS-R01 phase II trial evaluating neoadjuvant S-1 therapy for resectable pancreatic adenocarcinoma

**DOI:** 10.1038/s41598-022-14094-0

**Published:** 2022-06-15

**Authors:** Toru Nakamura, Tsuyoshi Hayashi, Yasutoshi Kimura, Hiroshi Kawakami, Kuniyuki Takahashi, Hirotoshi Ishiwatari, Takuma Goto, Masayo Motoya, Keisuke Yamakita, Yusuke Sakuhara, Michihiro Ono, Eiichi Tanaka, Makoto Omi, Katsuhiko Murakawa, Tomoya Iida, Tamaki Sakurai, Shin Haba, Takehiro Abiko, Yoichi M. Ito, Hiroyuki Maguchi, Satoshi Hirano, Hokkaido Pancreatic Cancer Study Group (HOPS), Toru Nakamura, Tsuyoshi Hayashi, Yasutoshi Kimura, Hiroshi Kawakami, Kuniyuki Takahashi, Hirotoshi Ishiwatari, Takuma Goto, Masayo Motoya, Keisuke Yamakita, Yusuke Sakuhara, Michihiro Ono, Eiichi Tanaka, Makoto Omi, Katsuhiko Murakawa, Tomoya Iida, Tamaki Sakurai, Shin Haba, Takehiro Abiko, Yoichi M. Ito, Hiroyuki Maguchi, Satoshi Hirano, Junpei Sasajima, Yohei Kitano, Nobuyuki Yanagawa, Kakuya Matsumoto, Satoshi Tanno, Masafumi Imamura, Masahiro Shitani, Minoru Takahashi, Hiroyuki Miyakawa, Fumitaka Nakamura, Yoshiyasu Ambo, Hirofumi Kamachi, Akinobu Taketomi

**Affiliations:** 1 Department of Gastroenterological Surgery II, Faculty of Medicine, Hokkaido University, N-15 W-7, Kita-Ku, Sapporo, Hokkaido 060-8638 Japan; 2 Center for Gastroenterology, Teine Keijinkai Hospital, 12-1-40 Maeda 1 Jo, Teine-ku, Sapporo, Hokkaido 006-0811 Japan; 3 Department of Surgery, Surgical Oncology and Science, School of Medicine, Sapporo Medical University, S1 W17, Chuo-ku, Sapporo, Hokkaido 060-8556 Japan; 4 Department of Gastroenterology and Hepatology, Faculty of Medicine, Hokkaido University, N-15 W-7, Kita-Ku, Sapporo, 060-8638 Japan; 5 Department of Medical Oncology, School of Medicine, Sapporo Medical University, S1 W17, Chuo-ku, Sapporo, Hokkaido 060-8556 Japan; 6 Division of Gastroenterology and Hematology/Oncology, Department of Medicine, Asahikawa Medical University, 1-1-1 Midorigaoka Higashi 2 Jo, Asahikawa, Hokkaido 078-8510 Japan; 7 Department of Gastroenterology and Hepatology, School of Medicine, Sapporo Medical University, S1 W17, Chuo-ku, Sapporo, Hokkaido 060-8556 Japan; 8 Division of Metabolism and Biosystemic Science, Department of Medicine, Asahikawa Medical University, 1-1-1 Midorigaoka Higashi 2 Jo, Asahikawa, Hokkaido 078-8510 Japan; 9 Department of Diagnostic and Interventional Radiology, Hokkaido University, N-15 W-7, Kita-Ku, Sapporo, 060-8638 Japan; 10 Department of Surgery, Hokkaido Gastroenterology Hospital, 1-2-10 Honcho 1 Jo, Higashi-ku, Sapporo, Hokkaido 065-0041 Japan; 11 Department of Surgery, Kushiro Red Cross Hospital, 21-14 Shineicho, Kushiro, Hokkaido 085-8512 Japan; 12 Department of Surgery, Obihiro Kosei General Hospital, 10-1 Nishi 14 Jominami, Obihiro, Hokkaido 080-0024 Japan; 13 Department of Gastroenterology, Muroran City General Hospital, 3-8-1 Yamatecho, Muroran, Hokkaido 051-8512 Japan; 14 Department of Gastroenterology, Steel Memorial Muroran Hospital, 1-45 Chiribetsucho, Muroran, Hokkaido 050-0076 Japan; 15 Department of Gastroenterology, NTT East Sapporo Hospital, S1 W15 Chuo-ku, Sapporo, Hokkaido 060-0061 Japan; 16 Department of Surgery, Asahikawa Red Cross Hospital, 1-1-1 Akebono 1 Jo, Asahikawa, Hokkaido 070-8530 Japan; 17 Biostatistics Division, Hokkaido University Hospital Clinical Research and Medical Innovation Center, Kita 14, Nishi 5, Kita-ku, Sapporo, Hokkaido 060-8648 Japan; 18 Department of Gastroenterology, Asahikawa Kosei Hospital, Asahikawa, Hokkaido Japan; 19 Department of Gastroenterology, Asahikawa Medical Center, Asahikawa, Hokkaido Japan; 20 Department of Gastroenterology, IMS Sapporo Digestive Disease Center General Hospital, Sapporo, Japan; 21 Sapporo Kyoritsu Gorinbashi Hospital, Sapporo, Japan; 22 Division of Bilio-Pancreatology, Department of Gastroenterology, Sapporo Kosei General Hospital, Sapporo, Japan; 23 Department of Surgery, Teine Keijinkai Hospital, Sapporo, Hokkaido Japan; 24 Department of Gastroenterological Surgery I, Hokkaido University Hospital, Sapporo, Hokkaido Japan

**Keywords:** Outcomes research, Chemotherapy, Cancer, Surgical oncology, Medical research, Clinical trials

## Abstract

Although neoadjuvant therapy (Nac) is recommended for high-risk resectable pancreatic cancer (R-PDAC), evidence regarding specific regimes is scarce. This report aimed to investigate the efficacy of S-1 Nac for R-PDAC. In a multicenter phase II trial, we investigated the efficacy of Nac S-1 (an oral fluoropyrimidine agent containing tegafur, gimeracil, and oteracil potassium) in R-PDAC patients. The protocol involved two cycles of preoperative S-1 chemotherapy, followed by surgery, and four cycles of postoperative S-1 chemotherapy. Two-year progression-free survival (PFS) rates were the primary endpoint. Overall survival (OS) rates and median survival time (MST) were secondary endpoints. Forty-nine patients were eligible, and 31 patients underwent resection following Nac, as per protocol (31/49; 63.3%). Per-protocol analysis included data from 31 patients, yielding the 2-year PFS rate of 58.1%, and 2-, 3-, and 5-year OS rates of 96.8%, 54.8%, and 44.0%, respectively. MST was 49.2 months. Intention-to-treat analysis involved 49 patients, yielding the 2-year PFS rate of 40.8%, and the 2-, 3-, and 5-year OS rates of 87.8%, 46.9%, and 33.9%, respectively. MST was 35.5 months. S-1 single regimen might be an option for Nac in R-PDAC; however, the high drop-out rate (36.7%) was a limitation of this study.

## Introduction

The National Comprehensive Cancer Network (NCCN) guidelines recommend upfront surgery for patients with resectable pancreatic ductal adenocarcinoma (R-PDAC) (clinical stage I or II), or neoadjuvant therapy (Nac) is also recommended for high-risk R-PDAC cases, such as those involving high levels of tumor marker, large primary tumors, weight loss, and so on^1^. However, evidence regarding specific regimens for Nac in R-PDAC is scarce, and participation in clinical trials is encouraged. A recent meta-analysis reported that the median overall survival (OS) of R-PDAC ranged from 12 to 25.3 months in upfront surgery, while the median OS associated with Nac group ranged from 10 to 50.2 months^2^. A separate meta-analysis based on intention-to-treat analyses has shown that patients with PDAC who received Nac had better long-term survival outcomes than patients who received upfront surgery (hazard ratio [HR] = 0.66; 95% confidence interval [CI]: 0.50–0.87, *P* = 0.003)^3^. In a large-scale propensity-score matched analysis, Nac with upfront surgery was associated with improved survival (median OS: 26 months vs. 21 months, HR = 0.72; 95% CI 0.50–0.87, *P* < 0.01)^4^. At present, at least eight randomized trials have investigated the use of Nac for R-PDAC. However, only two trials have reported long-term outcomes associated with preoperative treatment^5,6^. One of the trials was PREOPANC trial that showed survival benefit of preoperative chemoradiotherapy using gemcitabine with radiation (median OS: 16.0 vs. 14.3 months; HR = 0.78)^5^. The other trial was Prep-02/JSAP-05 trial that showed survival benefit of Nac using gemcitabine with S-1 (median OS: 36.7 vs. 26.6 months; HR = 0.72)^6^. Thus, there is sparse evidence with respect to the administration of Nac for R-PDAC and the optimal protocol.

The theoretical benefits of neoadjuvant therapy in R-PDAC are (a) early treatment of potentially metastatic disease, (b) identification of patients diagnosed with metastatic disease during treatment who can be spared surgical procedures that are unlikely to have survival benefit, and (c) delivery of chemotherapy and radiation to the primary tumor while it is in an intact, well-vascularized condition. In cases of R-PDAC, downsizing strategies to improve R0 resectability are not as important as they are in cases of borderline resectable PDAC or locally advanced PDAC. It should be noted that early treatment of potentially metastatic disease involves the same strategy as postoperative adjuvant therapy; however, only approximately 60% of patients with PDAC receive postoperative adjuvant therapy in the real world setting due to perioperative morbidity or early disease recurrence^7–9^. As such, the main purpose of Nac in the treatment of R-PDAC is the prevention of postoperative recurrence, which is consistent with postoperative adjuvant therapy. Preoperative treatment of R-PDAC does not need to reduce tumor size, as even if the local effect is weak, R0 resection is possible in cases of stable disease.

Given the advantages of Nac, S-1 (an oral fluoropyrimidine agent containing tegafur, gimeracil, and oteracil potassium) was selected for use in the present study; this regimen has been associated with relatively good outcomes as postoperative adjuvant therapy. The JASPAC01 trial has shown that the S-1 regimen is a superior adjuvant therapy to gemcitabine (Gem) in patients with R-PDAC (mortality HR = 0.57, 95% CI 0.44–0.72, *P* < 0.0001)^10^. In the same trial, disease recurrence in the liver was observed in 29% of patients in the Gem group and in 19% of patients in the S-1 group (*P* = 0.0016). Based on these findings, we hypothesized that S-1 might decrease the risk of micrometastasis, such as occult liver metastasis, in cases of R-PDAC. We conducted a multicenter single-arm phase II clinical trial to investigate the efficacy of S-1 Nac in patients with R-PDAC. This study is the first trial to focus on the prevention of R-PDAC recurrence using only a single oral agent for preoperative treatment.

## Results

### Patient characteristics

**Figure 1:**
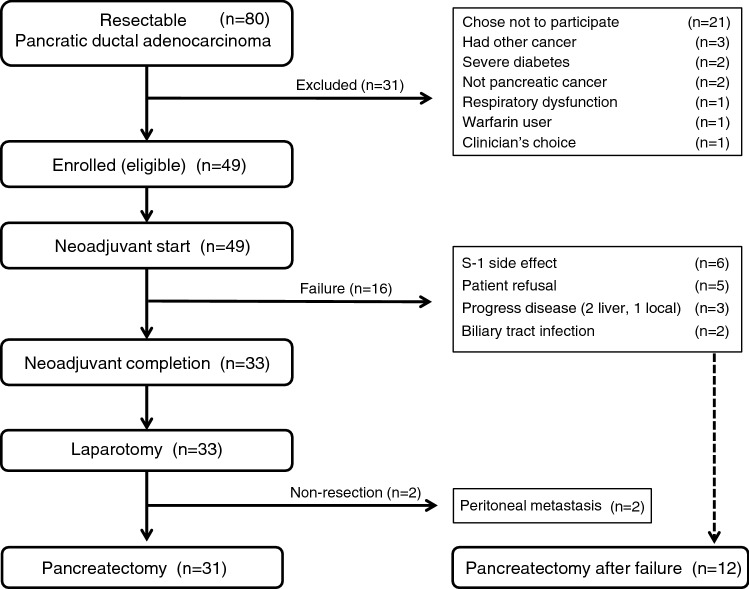
CONSORT diagram of the study flow. A total of 80 patients gave informed consent for the HOPS-R01 trial between January 2014 and October 2015 and were diagnosed with resectable pancreatic ductal adenocarcinoma by a central review of multidetector computed tomography. Neoadjuvant (Nac) S-1 at 80 mg/m^2^ per day was administered for 28 consecutive days followed by a 14-day rest (one cycle). The administration of S-1 was repeated every 6 weeks for two cycles. Of 49 patients who started Nac, 33 patients completed Nac and 31 patients received pancreatectomy. Of 16 Nac-failure patients, 12 patients underwent resection (pancreatectomy).

A total of 80 patients were diagnosed with R-PDAC by central review of multidetector computed tomography (MDCT) findings, under the informed consent. We enrolled 49 patients in this trial between January 2014 and October 2015. The CONSORT study flow summary is presented in Fig. 1. Patient demographic characteristics (n = 49) are summarized in Table 1.

**Table 1:** Baseline demographic characteristics of eligible patients (N = 49).

Characteristic	N (%)
**Sex**	
Male	21 (42.9)
Female	28 (57.1)
Age, median (range), years	71 (47–83)
**ECOG PS**	
0	47 (95.9)
1	2 (4.1)
**Tumor location**	
Head	36 (73.5)
Body	13 (26.5)
**Biliary drainage**	
Yes	19 (38.8)
No	30 (61.2)
Tumor size, median (range), mm	22 (7–58)
CA19-9, median (range), U/mL	45.3 (0.7–37,105.0)

ECOG PS, Eastern Cooperative Oncology Group performance status.

### Neoadjuvant therapy, adverse events, and disease response

Treatment-related adverse events (AEs) are shown in Table 2. Preoperative therapy was well-tolerated by hematological markers, however, S-1 AEs that directly resulted in protocol discontinuation were diarrhea, nausea, and vomiting. Of five patients who refused to continue Nac despite the absence of severe AEs, four patients opted for surgical resection, and one patient selected no other treatment. Among 49 eligible patients, a radiological partial response was observed in 11 (22.4%) patients, stable disease was observed in 33 (67.3%) patients, progressive disease was observed in three patients (two liver metastases, one local progression), and two patients underwent no evaluation. Changes from the baseline tumor size and CA19-9 levels are shown in Supplemental Fig. Se1.

**Table 2:** Adverse events* related to neoadjuvant S-1 therapy (N = 49).

Adverse event	Grade 1–2, n (%)	Grade 3, n (%)	Grade 4, n (%)
Anemia	13 (27)	1 (2)	0 (0)
Leukopenia	12 (25)	2 (4)	0 (0)
Neutropenia	10 (20)	4 (8)	0 (0)
Thrombocytopenia	1 (2)	1 (2)	0 (0)
Fatigue	6 (12)	1 (2)	0 (0)
Anorexia	12 (25)	2 (4)	0 (0)
Diarrhea	5 (10)	4 (8)	1 (2)
Mucositis oral	4 (8)	0 (0)	0 (0)
Nausea	3 (6)	2 (4)	0 (0)
Vomiting	2 (4)	2 (4)	0 (0)
Hyperpigmentation	6 (12)	0 (0)	0 (0)
Rash maculopapular	4 (8)	0 (0)	0 (0)
Biliary tract infection	–	3 (6)	0 (0)
Thromboembolic event	0 (0)	0 (0)	1 (2)

*Events were graded according to Common Terminology Criteria for Adverse Events (CTCAE) version 4.0.

### Surgical and pathological findings

After completing Nac, 33 patients proceeded to surgery (Fig. 1). Concurrently, 12 of 16 patients who experienced preoperative therapy failure were converted to surgery and received a pancreatectomy. The surgical and pathological findings of 31 patients who received pancreatectomy with Nac are shown in Table 3. Two patients received distal pancreatectomy with en bloc celiac axis resection (DP-CAR) due to suspected tumor involvement in the bifurcation of the celiac and splenic artery. Two patients had lymph node metastasis around the middle colic artery, which was diagnosed as extra regional lymph nodes (M1).

**Table 3:** Surgical and pathological outcomes in neoadjuvant complete patients (n = 31).

Surgical and pathological outcomes	N (%)
**Operative procedure**	
SSPPD	19 (61.3)
PD	1 (3.2)
PPPD	2 (6.5)
DP	7 (22.6)
DP-CAR	2 (6.5)
**Portal vein resection**	
No	25 (80.6)
Yes	6 (19.4)
No. of retrieved lymph nodes, median (range)	35 (7–102)
Blood loss, median (range), mL	440 (50–2150)
Operative time, median (range), min	428 (177–739)
Tumor size, median (range), cm	2.0 (0.5–4.4)
Lymph node metastasis	13 (41.9)
Portal vein invasion	5 (16.1)
Arterial invasion (celiac axis or SMA)	0
Plexus invasion	2 (6.5)
**Residual tumor (R)**	
R0	29 (93.5)
R1	2 (6.5)
**Pathological Stage (UICC 7th)**	
IA	5 (16.1)
IB	0
IIA	13 (41.9)
IIB	11 (35.5)
III	0
IV	2 (6.5)
**Pathological response (Evans classification)**	
I	12 (38.7)
IIa	12 (38.7)
IIb	6 (19.4)
III	1 (3.2)
IV	0

SSPPD, subtotal stomach-preserving pancreaticoduodenectomy; PD, pancreaticoduodenectomy; PPPD, pylorus-preserving pancreaticoduodenectomy; DP, distal pancreatectomy; DP-CAR, distal pancreatectomy with en-bloc celiac axis resection; SMA, superior mesenteric artery; UICC, Union for International Cancer Control.

The postoperative complications after pancreatectomy are shown in Table 4. Abdominal bleeding from the right gastric artery was found in a patient who received reoperation on postoperative day 3; however, there were no cases of either grade IV or V complications. The surgical and pathological results of patients receiving off-protocol resection (n = 12) are shown in Supplemental Tables Se1 and Se2. There were no cases of either grade IV or V complications in the off-protocol resections.

**Table 4:** Postoperative complications after resection (neoadjuvant therapy complete patients: n = 31).

Clavien-Dindo classification	I, n (%)	II, n (%)	IIIa, n (%)	IIIb, n (%)	IVa, n (%)	IVb, n (%)	V, n (%)
Pancreatic fistula	1 (3.2)	0 (0)	3 (9.7)	0 (0)	0 (0)	0 (0)	0 (0)
Delayed gastric emptying	0 (0)	0 (0)	1 (3.2)	0 (0)	0 (0)	0 (0)	0 (0)
Wound infection	1 (3.2)	3 (9.7)	0 (0)	0 (0)	0 (0)	0 (0)	0 (0)
Intra-abdominal abscess	0 (0)	2 (6.5)	2 (6.5)	0 (0)	0 (0)	0 (0)	0 (0)
Bile leakage	0 (0)	0 (0)	2 (6.5)	0 (0)	0 (0)	0 (0)	0 (0)
Abdominal bleeding	0 (0)	0 (0)	0 (0)	1 (3.2)	0 (0)	0 (0)	0 (0)
Cholangitis	0 (0)	1 (3.2)	0 (0)	0 (0)	0 (0)	0 (0)	0 (0)
Chylous ascites	0 (0)	1 (3.2)	0 (0)	0 (0)	0 (0)	0 (0)	0 (0)
Diarrhea	0 (0)	1 (3.2)	0 (0)	0 (0)	0 (0)	0 (0)	0 (0)
Portal vein embolism	0 (0)	1 (3.2)	0 (0)	0 (0)	0 (0)	0 (0)	0 (0)
Cerebral infarction	0 (0)	1 (3.2)	0 (0)	0 (0)	0 (0)	0 (0)	0 (0)
Intestinal bleeding	0 (0)	0 (0)	1 (3.2)	0 (0)	0 (0)	0 (0)	0 (0)
Postoperative hospital stay, days (range)	21 (12–67)						

Postoperative complications were analyzed in 31 patients who underwent pancreatectomy after complete neoadjuvant therapy.

### Adjuvant therapy

Of 31 patients who completed Nac followed by R0/R1 resection, 28 (90.3%) started S-1 adjuvant therapy and three patients did not (2 patients had poor performance status (PS) and one patient had bone metastasis before adjuvant therapy). Twenty-two patients completed all pre- and postoperative therapies as per study protocol (45% of 49 eligible patients and 71% of completed Nac patients). Meanwhile, among 12 patients who discontinued Nac, but received R0/R1 resection, 8 (66.7%) patients started adjuvant therapy and six patients completed it. Another four patients refused any chemotherapy.

### Survival analysis

**Figure 2:**
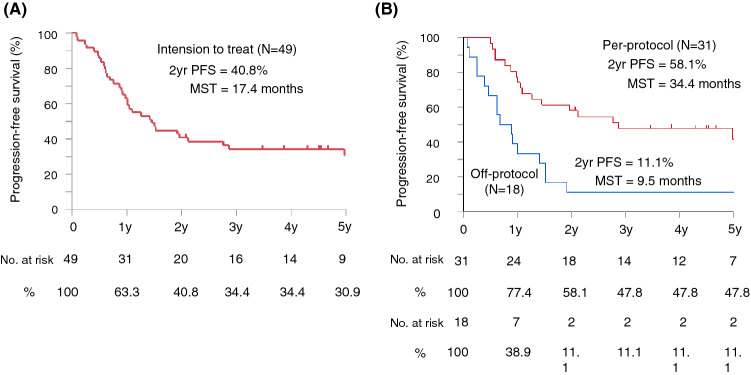
Kaplan–Meier survival curves of progression-free survival. (**A**) Intention-to-treat patients (n = 49). (**B**) Per-protocol patients (neoadjuvant treatment completion and tumor resection, n = 31) and off-protocol patients (neoadjuvant failure or probe laparotomy, n = 18). PFS, progression-free survival; MST, median survival time.

The 2-year progression-free survival (PFS) rate was 58.1% in 31 patients who completed Nac (per-protocol) followed by R0/R1 resection, and 40.8% in the intension-to-treat (ITT) analysis that included all 49 eligible patients (Fig. 2). The primary endpoint of this trial exceeded both the expected (48.6%) and threshold (28.9%) values in per-protocol patients; however, the outcome of the ITT analysis fell between the expected and threshold values. The 2-year PFS rate for 18 patients who did not complete Nac (off-protocol) was 11.1%. As reference data, 28 patients who had started adjuvant S-1 therapy after R0 resection are presented in Supplemental Fig. Se2. The 2-year relapse-free survival (RFS) and 5-year OS rates were 57.1% and 49.5%, respectively, and superior to those reported by the S-1 arm of JASPAC01 study (48.6% and 44.1% respectively).

**Figure 3:**
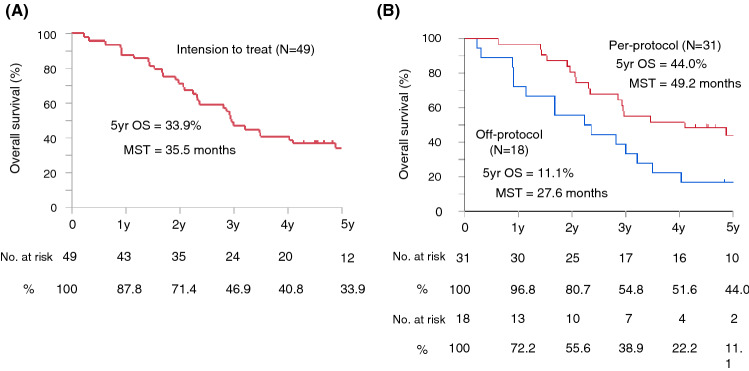
Kaplan–Meier survival curves for overall survival. (**A**) Intention to treat patients (n = 49). (**B**) Per-protocol patients (neoadjuvant treatment completion and tumor resection, n = 31) and off-protocol patients (neoadjuvant failure or probe laparotomy, n = 18). OS, overall survival; MST, median survival time.

The ITT values for the 2-, 3-, and 5-year OS rates of 49 eligible patients were 71.4%, 46.9%, and 33.9%, respectively. The median survival time (MST) was 35.5 months. The observed 2-, 3-, and 5-year OS rates for per-protocol patients (n = 31) were 80.7%, 54.8%, and 44.0%, respectively, and the MST was 49.2 months. The 2-, 3-, and 5-year OS rates for off-protocol patients (n = 18) were 55.6%, 38.9%, and 11.1%, respectively, and the MST was 27.6 months (Fig. 3). The OS values for patients with off-protocol resection (n = 12) were 66.7%, 50.0%, and 16.7%, respectively, with an MST of 34.9 months. The OS rates for patients not undergoing resection (n = 6) were 33.3%, 16.7%, 16.7%, respectively, with an MST of 15.6 months (Supplemental Fig. Se3). The median duration of follow-up was 35.5 months (range: 2.8–73.1 months) for all patients in this trial.

### Recurrence

First recurrence sites (includes patients with multi-site recurrences) are shown in Supplemental Table Se3. Of the per-protocol (Nac complete) resection patients (n = 31), 18 (58.1%) patients experienced disease recurrence; this rate was lower than that of the off-protocol (Nac failure) resections (10/12; 83.3%). The rate of distant metastasis was high among off-protocol resections (75% vs. 48.3%). Liver recurrence (disease progression) for per-protocol resections, off-protocol resections, and non-resection was found in 25.8%, 33.3%, and 50% of cases, respectively.

## Discussion

This trial showed that the 2-year PFS rate was 58.1% among 31 patients who completed Nac before receiving R0/R1 resection (per-protocol). In the per-protocol analysis, the primary endpoint yielded values that were better than expected. However, in the ITT analysis of 49 patients, including those who failed the protocol, the 2-year PFS rate was 40.8%, which was below the reference value. Trials of adjuvant therapy, such as the JASPAC01 study, have reported better results than those of the ITT analysis, due to the exclusion of patients with metastasis confirmed during surgery or those with poor PS after resection and at the time of adjuvant therapy initiation^3^. In fact, the present study subpopulation that met the JASPAC01 trial criteria showed relatively better survival (Supplemental Fig. Se2). A recent meta-analysis, including 18 studies that involved 857 patients, has reported that the MST associated with Nac for patients with R-PDAC was 18.2 months (range: 10–50.2 months)^2^. In our trial, the MST of all 49 patients in the ITT analysis was 35.5 months, suggesting that survival outcomes in the present study were better than those in the studies included in the meta-analysis.

At present, the evidence level for Nac in R-PDAC remains low and the optimal protocol remains unknown. At least eight trials have compared the role of neoadjuvant treatment with that of upfront surgery in the outcome of R-PDAC, and their preoperative protocols vary (Supplemental Table Se4). Of these, the PREOPANC trial (Gem followed by Gem combined with radiation, total 10 weeks regimen) was the first randomized phase III trial to publish findings on the use of Nac for PDAC (including both borderline resectable and resectable); a preplanned subgroup ITT analysis demonstrated superior OS for Nac patients with BR-PDAC (HR = 0.62, 95% CI 0.40–0.95, *P* = 0.029), but not for Nac patients with R-PDAC (HR = 0.96, 95% CI 0.64–1.44, *P* = 0.830)^5^. The subgroup settings for R-PDAC in the PREOPANC trial might be under-powered for analysis (65 Nac vs. 68 upfront surgery); furthermore, the median OS was 14.6 months in the Nac group, which was a disappointing finding. Nevertheless, the Prep-02/JSAP-05 trial (Gem plus S-1, two cycles, total 6 weeks regimen) reported in preliminary findings that a significant benefit was observed with Nac compared with upfront surgery (median OS: 36.7 vs. 26.6 months; HR = 0.72; 95% CI 0.55–0.94, *P* = 0.015)^6^. At present, no other trials have delivered high-quality evidence on the impact of Nac on R-PDAC compared with upfront surgery.

The estimated MST of the ITT analysis of our Nac S-1 monotherapy was 35.5 months, while the estimated MST in the ITT analysis of the Nac Gem plus S-1 (GS) patients in the Prep-02/JSAP-05 was 36.7 months, with no difference in survival. No data were available for comparing S-1 with GS in R-PDAC survival; however, the GEST study, which was a randomized three-arm phase III study for advanced pancreatic cancer, showed non-inferiority of S-1, but did not show superiority of GS to Gem alone for OS^11,12^. GS treatment was associated with a better tumor shrinkage effect than either Gem alone or S1 alone, although there was no advantage in survival. The treatment efficacy in the locally advanced disease had the advantage of GS in response rate, PFS, and OS over Gem alone^13^. The aim of Nac for R-PDAC is to prevent metastatic recurrence rather than local control, as R0 resection can be achieved even if no tumor shrinkage. In the present study, treatment failure due to local progression was found in a single case (resected after protocol failure), and the R0 resection rate among the resected cases was 93% (40/43), suggesting that preoperative adjuvant therapy with S-1 monotherapy can achieve local tumor control in R-PDAC.

In this study, Nac was well-tolerated from the viewpoint of hematological markers; however, the gastrointestinal toxicity rate was high (4/16; 25%). S-1 is associated with a risk of gastrointestinal toxicities, which are generally higher in Caucasian than in Asian populations due to differences in pharmacokinetics and pharmacodynamics^14^. The present trial included patients with an Asian background. However, pharmacokinetic and pharmacodynamic profile examination was outside the scope of the present study; thus, the exact reasons behind protocol failures associated with gastrointestinal toxicity remain unclear. We speculate that patients and physicians might be concerned about undergoing surgery when even minor gastrointestinal toxicities are present, as pancreatic resection is a major surgery and requires a cautious approach. An advantage of S-1 therapy is that it is a single oral agent that does not require intravenous treatment or frequent outpatient visits, thereby preserving medical resources. Nevertheless, the risk of gastrointestinal symptoms is high. In addition, this treatment might not be suitable for use in non-Asian populations.

The second leading factor for Nac failure in the present study was patient refusal to continue with treatment despite the absence of severe AEs. In fact, patients were more likely to select surgical resection than to continue with Nac. As surgical resection is the only curative treatment for R-PDAC, patients might be eager to avoid tumor progression, which would make them ineligible for surgery. In fact, patients who refused to continue Nac had a strong desire for resection; four of five patients proceeded to surgery after discontinuing Nac. One of the possibilities for the strong desire for resection may be the patients’ medical expenses for chemotherapy, and the other may be the inconvenience of outpatient chemotherapy. Future trials should present evidence to patients considering Nac discontinuation, and patients should be informed about the importance of completing Nac in the absence of AEs rather than immediately undergoing surgery.

In conclusion, S-1 neoadjuvant therapy for R-PDAC is safe and promising. S-1 monotherapy can be used as neoadjuvant therapy for patients with R-PDAC. However, well-designed, randomized controlled trials are required to better understand the safety profile and efficacy of this approach.

## Methods

### Trial design and treatment

This study was a multicenter, open-label, single-arm phase II trial of Nac S-1 in patients with R-PDAC, conducted by the Hokkaido Pancreatic Cancer Study Group (HOPS)^15,16^ (HOPS-R01 trial: University Hospital Medical Information Network Clinical Trials Registry [UMIN-CTR] number UMIN000013031, the date of first registration of the trial was 31/01/2014).

The Nac protocol involved two cycles of 40 mg of oral S-1 for a body-surface area of < 1.25 m^2^, 50 mg for a body-surface area of 1.25–1.5 m^2^, or 60 mg for a body-surface area of > 1.5 m^2^, administered twice per day for 28 consecutive days, followed by a 14-day rest period (one cycle). The length of Nac was 12 weeks, which was slightly shorter than the reported median PFS of S-1^12^, to balance the chance of resection with adequate tumor suppression and patient selection. After completing Nac, all patients underwent dynamic MDCT for restaging. All patients eligible for pancreatic resection underwent surgery 2–6 weeks after completing Nac. Patients with distant metastasis or locally advanced disease were excluded from this study, with further treatment at the discretion of the attending physician. All patients with R0/R1 surgical resection received four cycles of adjuvant S-1 therapy, which followed the same protocol as Nac. After completing therapy, all patients were followed up once every 3 months during the first 2 years, and once every 6 months from year 3 onwards. Tumor markers and MDCT of the chest/abdomen/pelvis or gadolinium ethoxybenzyl diethylenetriamine pentaacetic acid enhanced magnetic resonance imaging were monitored during the follow-up period, which ended 2 years after the enrollment of the last patient.

### Patient population

Central review of diagnostic imaging was performed according to the definition of the NCCN guidelines 2012 (version 2) by a radiologist (YS) and verified by a surgeon (TN) and a physician (HK). Inclusion criteria: cytologically or histologically confirmed PDAC; age ≥ 20 years; Eastern Cooperative Oncology Group PS of 0 to 1; sufficient dietary intake; and satisfactory levels of blood parameters (white blood cell count ≥ 3500/mm^3^ and < 12,000/mm^3^, neutrophil count ≥ 2000/mm^3^, hemoglobin ≥ 9.0 g/dL, platelet count ≥ 100,000/mm^3^, total bilirubin ≤ 2.0 mg/dL [≤ 3.0 mg/dL in patients with biliary drainage], aspartate transaminase and alanine aminotransferase ≤ 100 IU [≤ 150 IU in patients with biliary drainage], creatinine ≤ 1.2 mg/dL, and creatinine clearance estimate by Cockcroft-Gault equation ≥ 50 mL/min). Exclusion criteria: history of S-1 treatment; history of PDAC treatment; another simultaneous or metachronous (within 3 years) cancer; current use of flucytosine, phenytoin or warfarin; watery diarrhea; pulmonary fibrosis or intestinal pneumonia; and confirmed or suspected pregnancy in women.

Preoperative treatment-related AEs were assessed using the Common Terminology Criteria for Adverse Events (version 3.0). Surgical resection was performed by laparotomy and regional lymph node dissection was required. Resection of the portal vein/superior mesenteric vein was allowed. A DP-CAR due to suspected tumor involvement in tumor proximity to the bifurcation of the celiac and splenic artery was allowed^17^. Surgical morbidity was evaluated based on the Clavien-Dindo classification^18^. Pancreatic fistula was evaluated according to the classification of the International Study Group of Pancreatic Surgery^19^. Pathology findings were assessed by pathologists at each participating hospital, using Union for International Cancer Control -TNM version 7 and the Evans classification^20^. The study protocol was approved by the Institutional Review Board of each participating hospital and the study adhered to the Declaration of Helsinki (IRB No. 013–0059, Institutional Review Board of Hokkaido University Hospital, the date of first registration was 14/02/2014, the registration number was HOPS-R01-01).

### Endpoints and statistical analysis

The primary endpoint was 2-year PFS. Secondary endpoints included OS, resection, and response rates, measured according to RECISTv1.1, pathological outcomes, preoperative treatment-related AEs, and surgical morbidity rate. PFS was defined as the time from registration with the trial to the date of first recurrence or disease progression, either local, distant, or both, whichever occurred first. Recurrence was defined as a radiologically rather than elevation of CA19-9. OS was defined as time from registration to the date of death from any cause and censored on the date of the final confirmation of survival for surviving patients. It was estimated using the Kaplan–Meier method.

To calculate the desired sample size for the present study, the threshold and expected values of the 2-year PFS rates were set at 29% and 48%, respectively. These estimates were based on the JASPAC01 study findings, where 2-year RFS was 48% in the S-1 group and 29% in the Gem group in an adjuvant setting^10^. Given this threshold (29%) and expected 2-year PFS (48%), the sample size was calculated as 46, based on the Southwest Oncology Group one arm binomial tool, with a significance level of 0.025 and power of 80%. In anticipation of loss to follow-up, we expected to enroll 50 patients in the present study**.**

## Supplementary Information

Supplementary Information 1.

Supplementary Information 2.

Supplementary Information 3.
