# Supplementary material for: HOPS-R01 phase II trial evaluating neoadjuvant S-1 therapy for resectable pancreatic adenocarcinoma
Source: Sci Rep. 2022 Jun 15;12:9966. doi: 10.1038/s41598-022-14094-0 (PMC9200853; doi:10.1038/s41598-022-14094-0)
Supplement: Supplementary file 1 — Supplementary Information 1. [file 41598_2022_14094_MOESM1_ESM.docx]

Supplementary information

| **TABLE e1.** Surgical and Pathological Results after Failure of Neoadjuvant Therapy in 12 Patients | |
| --- | --- |
| **Surgical and Pathological Outcomes** | **N (%)** |
| Operative procedure |  |
| SSPPD | 7 (58.3) |
| DP | 4 (33.3) |
| TP | 1 (8.3) |
| Portal vein resection |  |
| No | 8 (66.7) |
| Yes | 4 (33.3) |
| No. of retrieved lymph nodes, median (range) | 36 (14–64) |
| Blood loss, median (range), mL | 590 (512–1820) |
| Operative time, median (range), min | 421 (175–667) |
| Tumor size, median (range), cm | 2.9 (1.3–5.5) |
| Lymph node metastasis | 10 (83.3) |
| Portal vein invasion | 4 (33.3) |
| Arterial invasion (celiac axis or SMA) | 0 |
| Plexus invasion | 5 (41.7) |
| Residual tumor (R) |  |
| R0 | 11 (91.7) |
| R1 | 1 (8.3) |
| Pathological Stage (UICC 7^th^) |  |
| IA | 0 |
| IB | 0 |
| IIA | 2 (16.7) |
| IIB | 9 (75.0) |
| III | 0 |
| IV | 1 (8.3) |
| Pathological response (Evans Classification) |  |
| I | 7 (58.3) |
| IIa | 3 (25.0) |
| IIb | 2 (16.7) |
| III | 0 |
| IV | 0 |
| Abbreviations: SSPPD, subtotal stomach-preserving pancreaticoduodenectomy; DP, distal pancreatectomy; TP, total pancreatectomy; SMA, superior mesenteric artery; UICC, Union for International Cancer Control. | |

| **TABLE e2.** Postoperative Complications after Resection (Neoadjuvant Therapy Failure Patients: n = 12) | | | | | | | |
| --- | --- | --- | --- | --- | --- | --- | --- |
| **Clavien-Dindo classification** | **I, n (%)** | **II, n (%)** | **IIIa, n (%)** | **IIIb, n (%)** | **IVa, n (%)** | **IVb, n (%)** | **V, n (%)** |
| Pancreatic fistula | 1 (8.3) | 0 (0) | 0 (0) | 0 (0) | 0 (0) | 0 (0) | 0 (0) |
| Delayed gastric emptying | 0 (0) | 0 (0) | 1 (8.3) | 0 (0) | 0 (0) | 0 (0) | 0 (0) |
| Wound infection | 1 (8.3) | 1 (8.3) | 0 (0) | 0 (0) | 0 (0) | 0 (0) | 0 (0) |
| Liver abscess | 0 (0) | 0 (0) | 1 (8.3) | 0 (0) | 0 (0) | 0 (0) | 0 (0) |
| Paralytic ileus | 0 (0) | 1 (8.3) | 0 (0) | 0 (0) | 0 (0) | 0 (0) | 0 (0) |
| Diarrhea | 0 (0) | 1 (8.3) | 0 (0) | 0 (0) | 0 (0) | 0 (0) | 0 (0) |
| Anastomotic leakage (colon) | 0 (0) | 0 (0) | 0 (0) | 1 (8.3) | 0 (0) | 0 (0) | 0 (0) |
| Pulmonary embolism | 0 (0) | 1 (8.3) | 0 (0) | 0 (0) | 0 (0) | 0 (0) | 0 (0) |
| Postoperative hospital stay, day (range) | 29.5 (10-115) | | | | | | |
| Postoperative complications were analyzed in 31 patients who underwent pancreatectomy after complete neoadjuvant therapy. | | | | | | | |

| **TABLE e3.** First Site of Recurrence (Disease Progression) | | | |
| --- | --- | --- | --- |
| **Site of progression*** | **Resection after Nac completion (n = 31)**  **n (%)** | **Resection after Nac failure (n = 12)**  **n (%)** | **Non resection**  **(n=6)**  **n (%)** |
| Recurrence patients | 18 | 10 | 6 |
| Local | 5 (16.1) | 1 (8.3) | 1 (16.7) |
| Lymph node | 4 (12.9) | 0 (0) | 0 (0) |
| Distant metastasis | 15 (48.3) | 9 (75.0) | 5 (83.3) |
| Liver | 8 (25.8) | 4 (33.3) | 3 (50.0) |
| Peritoneum | 3 (6.5) | 3 (25.0) | 1 (16.7) |
| Lung | 2 (6.5) | 2 (16.7) | 1 (16.7) |
| Bone | 1 (3.2) | 0 (0) | 0 (0) |
| Ovary | 1 (3.2) | 0 (0) | 0 (0) |
| *First recurrence sites (includes patients with multi-site recurrences) | | | |

| **TABLE e4.**  Randomized trials compared the neoadjuvant treatment with upfront surgery in the R-PDAC | | | |
| --- | --- | --- | --- |
| **Trial** | **Neoadjuvant treatment** | **Phase** | **Status** |
| NEOPAC: NCT01521702 | Gem plus oxaliplatin | III | On going |
| NEOPA: NCT01900327 | Gem with radiation | III | Terminated  (Recruitment failure) |
| Prep-02/JSAP-05: UMIN000009634 | Gem plus S-1 | II/III | Completed |
| PREOPANC: NTR3709 | Gem followed by Gem combined with radiation | III | Completed |
| NEOPAFOX: NCT02172976 | FOLFIRINOX | II/III | On going |
| NEONAX: NCT02047513 | Gem plus nab-paclitaxel | II | On going |
| NorPACT-1: NCT02919787 | 5-FU plus irinotecan plus oxaliplatin | II/III | On going |
| CISPD-1: NCT03750669 | equential use of Gem plus nab-paclitaxel and mFOLFIRINOX | II | On going |
| NCT; ClinicalTrials.gov identifier  UMIN; University Hospital Medical Information Network Clinical Trials Registry  NTR ; Netherlands Trial Register | | | |
