# Supplementary material for: HOPS-R01 phase II trial evaluating neoadjuvant S-1 therapy for resectable pancreatic adenocarcinoma
Source: Sci Rep. 2022 Jun 15;12:9966. doi: 10.1038/s41598-022-14094-0 (PMC9200853; doi:10.1038/s41598-022-14094-0)
Supplement: Supplementary file 2 — Supplementary Information 2. [file 41598_2022_14094_MOESM2_ESM.pptx]

## Slide 1
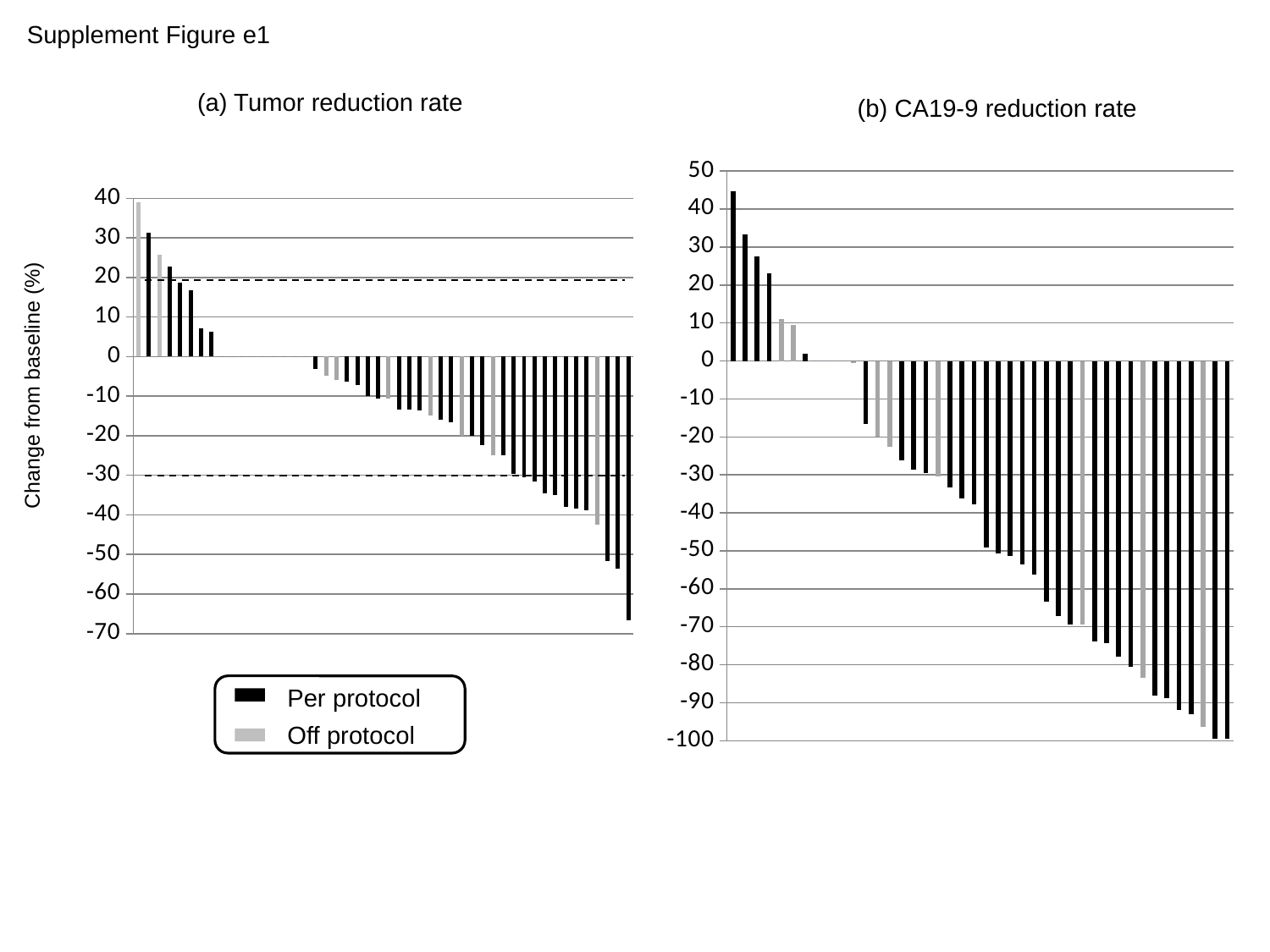

Supplement Figure e1
(a) Tumor reduction rate
(b) CA19-9 reduction rate
### Chart
| Category | CA19-9
reduction rate |
|---|---|
### Chart
| Category | Tumor
reduction rate |
|---|---|Change from baseline (%)
Per protocol
Off protocol

## Slide 2
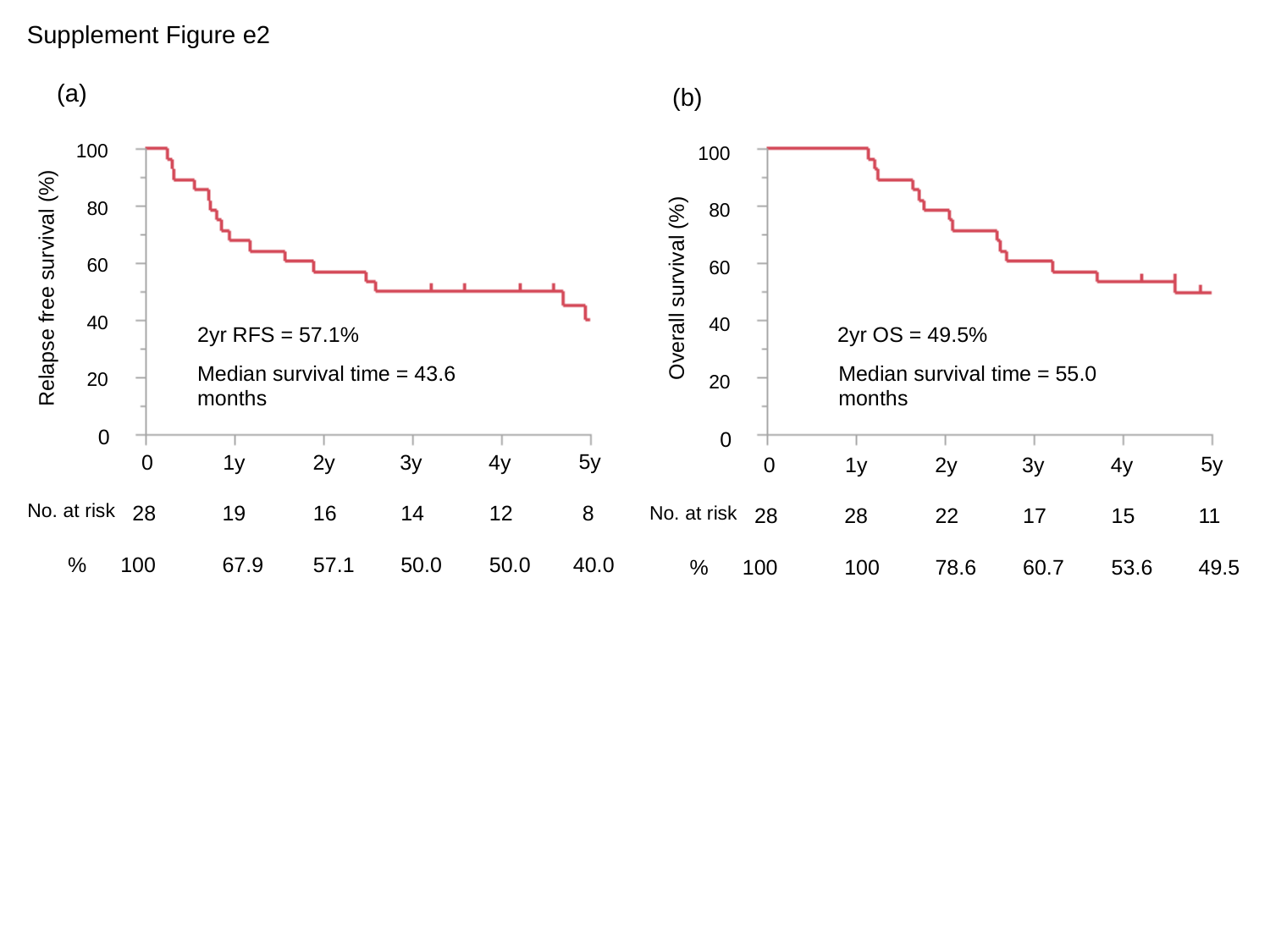

Supplement Figure e2
(a)
(b)
100
100
80
80
60
60
Relapse free survival (%)
Overall survival (%)
40
40
2yr RFS = 57.1%
2yr OS = 49.5%
Median survival time = 43.6 months
Median survival time = 55.0 months
20
20
0
0
5y
0
1y
2y
3y
4y
5y
0
1y
2y
3y
4y
No. at risk
28
19
16
14
12
 8
No. at risk
28
28
22
17
15
11
%
100
67.9
57.1
50.0
50.0
40.0
%
100
100
78.6
60.7
53.6
49.5

## Slide 3
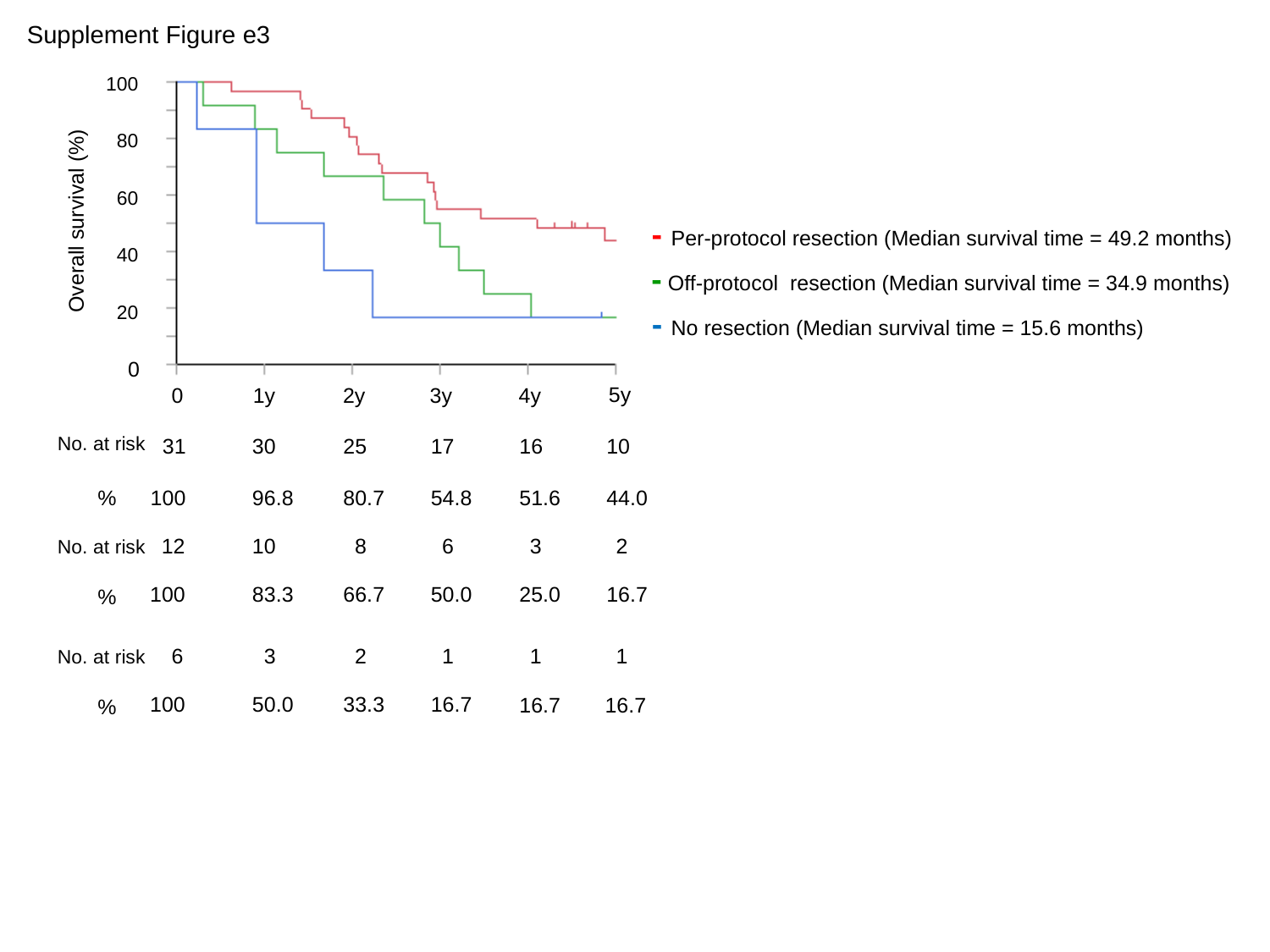

Supplement Figure e3
100
80
60
Overall survival (%)
- Per-protocol resection (Median survival time = 49.2 months)
40
- Off-protocol resection (Median survival time = 34.9 months)
20
- No resection (Median survival time = 15.6 months)
0
5y
0
1y
2y
3y
4y
No. at risk
31
30
25
17
16
10
%
100
96.8
80.7
54.8
51.6
44.0
12
10
8
6
3
2
No. at risk
100
83.3
66.7
50.0
25.0
16.7
%
6
3
2
1
1
1
No. at risk
100
50.0
33.3
16.7
16.7
16.7
%
