# Supplementary material for: HOPS-R01 phase II trial evaluating neoadjuvant S-1 therapy for resectable pancreatic adenocarcinoma
Source: Sci Rep. 2022 Jun 15;12:9966. doi: 10.1038/s41598-022-14094-0 (PMC9200853; doi:10.1038/s41598-022-14094-0)
Supplement: Supplementary file 3 — Supplementary Information 3. [file 41598_2022_14094_MOESM3_ESM.docx]

**Supplementary Figure Legends**

**Supplement Figure e1**

Waterfall plots of tumor reduction (a), and CA19-9 reduction (b) by preoperative therapy are shown. Tumor reduction rate was decrease in the sum of diameters of tumor, taking as reference the baseline sum diameters of tumor by calculate as follows, 100 x (baseline tumor diameters - after preoperative treatment tumor diameters) / (baseline tumor diameters). CA19-9 reduction rate was calculated as follows, 100 x (baseline CA19-9 value - after preoperative CA19-9 value) / (baseline CA19-9 value). The black bar showed per protocol patients and the gray bar showed off protocol patients.

**Supplement Figure e2**

Twenty-eight patients who had been start adjuvant S-1 therapy after R0 resection, which had the same conditions as the S-1 arm of JASPAC01, were extracted, and analyzed. (a) Recurrence free survival (RFS). (b) Overall survival (OS).

**Supplement Figure e3**

Comparison of overall survival of patients with per-protocol patients (n=31) and patients with off-protocol resection (n=12) and patients with not undergoing resection (n=6) are shown. Resection had a certain effect on survival, even in off-protocol patients.
